# A Retrospective Dosimetric Study of Radiotherapy Patients with Left-Sided Breast Cancer; Patient Selection Criteria for Deep Inspiration Breath Hold Technique

**DOI:** 10.3390/cancers11020259

**Published:** 2019-02-22

**Authors:** Mikaela Dell’Oro, Eileen Giles, Amy Sharkey, Martin Borg, Caroline Connell, Eva Bezak

**Affiliations:** 1 Cancer Research Institute and School of Health Sciences, University of South Australia, Adelaide, SA 5001, Australia; eileen.giles@unisa.edu.au (E.G.); Eva.Bezak@unisa.edu.au (E.B.); 2 GenesisCare, Adelaide, SA 5000, Australia; Amy.Sharkey@genesiscare.com (A.S.); hughes46@tpg.com.au (M.B.); Caroline.Connell@genesiscare.com (C.C.); 3 Department of Physics, University of Adelaide, Adelaide, SA 5005, Australia

**Keywords:** Deep inspiration breath hold, DIBH, left-sided breast cancer, total lung volume, TLV, chest wall separation, CWS, selection criteria

## Abstract

Background: Several studies have investigated cardiac dose reduction when utilizing the deep inspiration breath hold (DIBH) technique in patients undergoing radiotherapy for left-sided breast cancer. This paper aims to recommend potential selection criteria based on a retrospective single institute study of free breathing (FB) and DIBH computed tomography (CT) simulation planning scans. Methods: Dosimetric comparisons were performed retrospectively for 20 patients correlating the dose reduction and patient anatomical factors (anatomical variation of chest shape, chest wall separation, total lung volume (TLV) and others). Results: Paired t-tests demonstrated significant cardiac dose reduction for most patients but not all. Minimal cardiac dose reduction was observed for three patients using their DIBH plan, with one patient receiving a higher dose. Linear regression analysis identified a positive correlation between the patient’s TLV (on the FB CT simulation scan) and the magnitude of dosimetric benefit received (0.4045 R^2^). Conclusion: The TLV measured on a FB plan could potentially be utilised to predict cardiac exposure and assist with patient selection for DIBH. This is important in resource allocation, as DIBH may be unnecessarily recommended for some patients with little dosimetric benefit.

## 1. Introduction

Left-sided breast cancer radiotherapy results in a higher incidence of ischaemic heart disease (IHD) and premature cardiac mortality [1]. The heart and coronary arteries are located more anteriorly on the left side of the chest wall, receiving considerably more radiation dose due to geometrical changes during respiration compared to treatment for the right breast. There is evidence that radiation-induced IHD is directly proportional to over-irradiation of the left anterior descending artery (LAD) [2]. Managing respiratory motion of the chest wall should therefore be a consideration in minimizing dose to cardiac structures for patients with left-sided breast cancer. Respiratory gated radiotherapy is a technique used to manage geometric changes of the patient’s anatomy during respiration, delivering radiation whilst the patient is in a static phase of the respiratory cycle. If a deep inspiration phase is used, the fractional volume of the lung within the field decreases as the tissue expands compared to the conventional free breathing (FB) technique; this is known as deep inspiration breath hold (DIBH) respiratory gating [3]. DIBH reduces the heart dose by displacing it from the radiation field and maximizing the distance between the planning target volume (PTV) and cardiac structures whilst reducing target motion [4,5]. Throughout this paper, the corresponding computed tomography (CT) plans for free breathing and DIBH will be respectively referred to as CT_FB_ and CT_DIBH_ plans. 

It is generally indicated across the literature that all left-sided breast cancer patients benefit from the DIBH technique [2,4,5,6,7,8,9,10,11,12,13,14,15,16,17]; however, not all patients achieve the same benefit. A meta-analysis performed by Latty et al. [3] reviewed 18 studies which demonstrated a relative reduction of mean dose (D_mean_) to the heart ranging from 26.2% to 75%. As most reported papers present averages rather than patient-by-patient analyses, outliers go unidentified [4,14,17]. After reviewing the existing literature, Latty et al. [3] concluded that gaps existed in determining selection criteria to predict patients who benefit the most from DIBH. This suggests that future planning studies should investigate the use of anatomical parameters for patient selection. Only a few published papers reported on selection criteria for determining the magnitude of dosimetric benefit the DIBH technique offers individual patients [8]. This is especially important for patients with unfavorable chest wall shape (i.e., variations of pectus excavatum or pectus carinatum) [18,19]. Furthermore, patient selection for the DIBH technique varies across the literature from consultation to post-planning evaluation. Studies have selected patients based on age, presence of heart in the treatment field, ability to breath-hold (BH) for a predetermined length of time, anatomical parameters (i.e., maximum heart distance (MHD)) and/or post-planning comparison of the respective Dose Volume Histograms (DVH) for the CT_FB_ and CT_DIBH_ plans [3]. Joo et al. [10], Sung et al. [12] and Wang et al. [16] retrospectively analyzed eligibility through the implementation of a devised selection criteria, formulated from previous studies or departmental protocols. These studies trialed selection criteria across their samples prior to enrolment and therefore the findings cannot be as easily generalized as patients were not consecutively inducted into their respective studies [10,12,16]. Furthermore, the patients who were selected at the physician’s discretion received twice the CT simulation radiation dose as they had to be simulated in both FB and DIBH technique. This has led to concerns in the increase of workload and resources, so the technique is not as routinely implemented. 

Two-dimensional anatomical parameters measured on a CT simulation scan have been demonstrated to have a role in predicting the magnitude of dose received by the cardiopulmonary structures [20]. Two papers by Rochet et al. [21] and Register et al. [22] analyzed anatomical factors that could influence organ at risk (OAR) sparing. At the time of this review, only one study conducted by Johansen et al. [23] and a conference presentation abstract by Chilukuri et al. [24] had investigated the impact of anatomical variation between chest shapes and the benefit this may have on the use of DIBH (in terms of D_mean_). Johansen et al. [23] concluded that the variability of benefit depends on chest shape. However, all anatomical parameters investigated proved inconclusive, potentially due to small sample size. Chilukuri et al. [24] in their abstract, concluded chest wall shape could effectively identify patient suitability for DIBH, proposing patients with ‘flat’ chest walls should have cardiac doses investigated using the technique. In searching the published literature, no internationally recommended set of measurable parameters that define anatomical variations in patients’ thoracic shape were identified. Standard measurements for chest shape usually describe congenital abnormalities of the thoracic cage. These congenital abnormalities are recognized to have implications on the anatomical location of cardiopulmonary and vascular structures [18]. For example, an unfavorable chest wall shape can compromise PTV coverage in a small percentage of cases where the heart moves anteriorly with the thorax contents negating the effect of DIBH [25]. Despite convincing published data, gaps still exist in terms of identifying optimal candidates by their anatomical traits for the DIBH technique. The gaps identified in the literature justify the methodology utilised in this work with the aim to develop selection criteria for left-sided breast cancer patients based on comparing retrospective CT_FB_ and CT_DIBH_ plans. 

## 2. Material and Methods

The study design was a single institutional quantitative retrospective analysis of dosimetric data from 20 left-sided breast cancer patients, treated at the Adelaide Radiotherapy Centre (ARC), Adelaide, SA, Australia. All 20 patients were enrolled in a pilot study between May and September 2015 and completed two CT simulation scans: one in FB and one in DIBH using the Active Breathing Coordinator (ABC) device (Elekta AB, Stockholm, Sweden) or voluntary BH. In this investigation, the patient plans using either voluntary BH or the ABC device are referred to as the CT_DIBH_ plans. The patients were not classified as different cohorts per BH method (i.e., ABC or voluntary BH) because the study aimed to investigate the benefit of the technique itself and not the effect of the equipment. In order to investigate dosimetric advantages of DIBH, clinically acceptable CT_FB_ plans of all 20 patients were dosimetrically compared to existing CT_DIBH_ plans (see Table 1).

The treatment planning system (TPS) used in this work was Pinnacle^3^ V9.8 (Philips Radiation Oncology Systems, Fitchburg, WI, USA). Relevant dose-volume data was retrieved from DVHs produced by the TPS. MIM V6.6.2 software program (MIM Software, Inc., Cleveland, OH, USA) was used to extract data for inter- and intra-observer testing. GraphPad Prism 7 V7.02 software program (GraphPad Software Inc., San Diego, CA, USA) and Excel Microsoft (Excel 2010, Microsoft, Redmond, WA, USA) were used for data analysis.

A sample size of 20 was statistically calculated to show significant results using a post-hoc power calculation performed by the G*Power statistical analysis program (version 3.1.9.2, Heinrich-Heine-Universität, Düsseldorf, Germany, Faul et al. [26]). Aiming for a large effect size of 0.6 with a probability of type I error of 0.05, the power of the study allowed detection of existing relationships amongst variables. A *p*-value of less than 0.05 was considered statistically significant when performing two tailed paired t-tests between CT_DIBH_ and CT_FB_ plans/groups (i.e., 5% confidence interval limit). 

This study used convenience sampling as the data was retrospectively available. All patients were referred for adjuvant radiotherapy after surgery (mastectomy or breast-conserving surgery (BCS)) and were selected according to a broad inclusion criterion.

Inclusion Criteria:

- Women diagnosed with left-sided breast cancer requiring radiotherapy to the breast, chest wall with/without supraclavicular region, without internal mammary chain irradiation
- Consented to undergo CT simulation scans in DIBH and FB with their data permitted for future use in research
- Planned for treatment between May and September 2015
- Completed treatment at ARC.

Exclusion Criteria:

- Did not have both CT simulation scans (FB and DIBH) completed.

Several analyses were performed on the data collected to achieve the study aims. The distribution of data was firstly checked for normality, assuming a Gaussian distribution. The level of statistical significance across the tables and figures in this work are as follows unless specifically indicated; * *p*-value < 0.05, ** *p*-value < 0.01, *** *p*-value < 0.001, **** *p*-value < 0.0001.

With parametric assumptions satisfied between the two treatment groups, *t*-tests were performed to determine if the CT_DIBH_ plan provided a dose reduction to OAR. 

Anatomical factors were investigated using correlation analysis to quantify the relationship between the independent (dose difference (Δ)) and dependent variables (central lung distance, chest wall separation, maximum heart distance, body mass index (BMI), Haller Index (HI) and lung/s volume). 

Correlation analysis and linear regression tests were used to investigate any significant relationship between anatomical parameters and the dose or relative dose difference (Δ). The R^2^ value was calculated from Pearson’s R correlation coefficient and represents the fraction of the variance in the two variables (independent and dependent) that is ‘shared’.

## 3. Ethics Approval and Consent to Participate

Ethics approval was obtained from the Human Ethics Committee Review Group at the University of South Australia on 30th September 2016 (Application number: 0000035832). Participants signed an agreement stating their retrospective data may be used for future studies and publications under the condition that all information remains anonymous. 

## 4. Delineation of Regions of Interest

All target volumes and OARs in this study were contoured in accordance with the Radiation Therapy Oncology Group (RTOG) Breast and Cancer Contouring Atlas (2009) and clinical site protocols. The contouring of the CT_DIBH_ plans was performed and checked in the TPS by a range of physicians as part of pre-treatment quality assurance (QA) checks. Similarly, the CT_FB_ OAR contours were delineated by a Radiation Therapist (RT) according to centre protocols and verified by a senior RT, ensuring that all regions of interest (ROI) were contoured consistently.

The clinical target volume (CTV) was defined as the tumour bed including subclinical malignant disease (illustrated on the scans by surgical clips and/or surgical notes) according to ICRU Report 62 (1999) [27]. The PTV was expanded from the CTV to encompass the entire breast tissue with an anterior margin of 5 mm from the skin edge used to evaluate the target coverage.

## 5. Inter- and Intra-Observer Testing of LAD

The LAD structure is not regularly contoured and is therefore subject to increased inter-observer error impacting the reliability and validity of the extracted dosimetric data. The structure was contoured as recommended by Feng et al. [28]. Inter- and intra-observer testing of LAD contouring was performed in this work to increase reliability. Inter-observer tests were conducted by comparing the LAD contours of 10 randomly selected patients between the RT, senior RT and radiation oncologist at the clinical site. Similarly, intra-observer tests were conducted comparing the LAD contours of 10 randomly selected patients drawn two months apart by the RT. 

## 6. Treatment Planning and Evaluation

A step-and-shoot three-dimensional conformal radiotherapy (3DCRT) forward planning approach was used to produce a medial and lateral tangential opposing beam pair. The non-diverging beam edges traversed the lung and in some cases the heart to achieve homogenous dose distribution and dose objectives in Table 1. Minor variations existed between dose conformity, target dose coverage, maximum dose, beam energy and geometry to achieve equal clinical plan dosimetry.

The plans used 6 MV or 10 MV photon beam energies (or a combination) depending on the patient’s PTV size to achieve uniform coverage with the prescribed dose. The isocentre was prescribed 50 Gy or 42.4 Gy radiation dose delivered in 25 fractions or 16 fractions respectively. Wedges, multi-leaf collimators (MLC) and segmented beam weightings were optimized to achieve homogeneity across the target volumes. The CT_DIBH_ plans were segmented to span over 2–3 breath holds therefore the number of segments was replicated in the respective CT_FB_ plans. A collapsed cone convolution algorithm and a dose grid of 0.3 × 0.3 × 0.3 cm^3^ was used for dose calculations.

The CT_DIBH_ plans were clinically delivered and accepted for this study. The corresponding CT_FB_ treatment plans were optimized in the TPS to achieve comparable target dose coverage whilst minimizing OAR doses (see Table 1). The CT_FB_ plans were planned for the best clinical outcome, while attempting to keep all beam parameters as similar as possible to the CT_DIBH_ plan (achieving the planning goals of the clinical site).

All CT_FB_ plans were clinically acceptable as per Table 1. A sample of 40% of CT_FB_ plans were confirmed by a senior RT as clinically treatable plans, indicating minimal difference in achieving the same clinical objectives as the CT_DIBH_ plans. 

## 7. Patient Data Collection

The median patient age was 49 years (range: 33–71 years). All 20 patients underwent FB and DIBH scans, 10 patients completing voluntary BH while the other 10 underwent ABC equipped BH. Of the 20 patients, six were treated with the hypo-fractionated regime of 42.4 Gy in 16 fractions and 14 with the conventional 50 Gy in 25 fractions. The patients also ranged in staging, elective treatment and disease classification. 

The patient data collected in Table 2 was believed to contribute to the impact of treatment including dosimetric parameters evaluated for both (CT_FB_ and CT_DIBH_) plans in the TPS for each of the 20 patients. These dose reporting parameters were chosen based on the frequency of reporting in the literature and clinical site protocols. Anatomical parameter measurements were performed according to the definitions discussed below on all CT_FB_ and CT_DIBH_ plans for each of the 20 patients. In addition to anatomical parameters, other parameters such as HI and BMI, were acquired.

## 8. Haller Index

**Figure 1:**
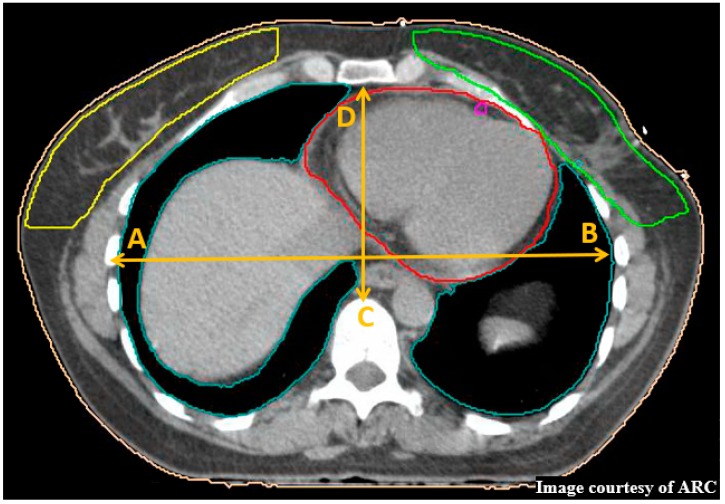
The Haller Index (HI) demonstrated on patient 17’s free breathing (FB) plan as the ratio between transverse diameter of the chest (length A→B) and the shortest distance between the sternum and vertebrae (length C→D) (Image Courtesy of ARC).

HI is defined as the ratio between transverse diameter of the chest and the shortest distance between the sternum and vertebrae [19]. The HI is measured at the deepest aspect of the thoracic curve, with a normal chest HI ratio calculated as 2 or less. This index was investigated to identify chest types and if this impacted the effects of DIBH treatment (Figure 1).

## 9. Maximum Heart Distance

**Figure 2:**
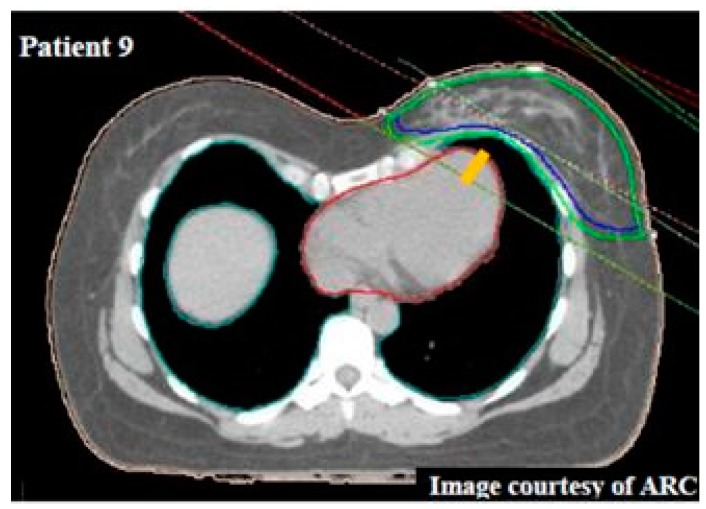
Maximum heart distance (MHD) (yellow) measured for patient 9 (deep inspiration breath hold (DIBH) plan) was 1.2 cm demonstrating the close proximity of the heart (red) in relation to the anterior left chest wall (clinical (blue) and planning (green) target volume (image courtesy of ARC)).

The maximum heart distance (MHD) is measured on the CT slice with the thickest section of heart contained within the field and is defined as the distance between the anterior cardiac contour crossing over the posterior edge of the tangential fields (Figure 2) [1]. MHD shortens with DIBH as inspiration maximizes the distance between the chest wall and heart, reducing the fractional heart volume receiving higher doses as its anterior surface is further away from the beam edge [29].

## 10. Central Lung Distance and Chest Wall Separation

**Figure 3:**
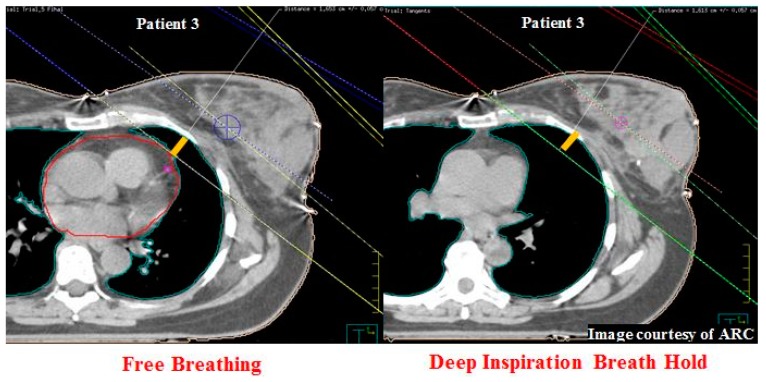
Central lung distance (CLD) (yellow) measured across FB (left) and DIBH (right) plans for patient 3 was identical (Image Courtesy of ARC).

**Figure 4:**
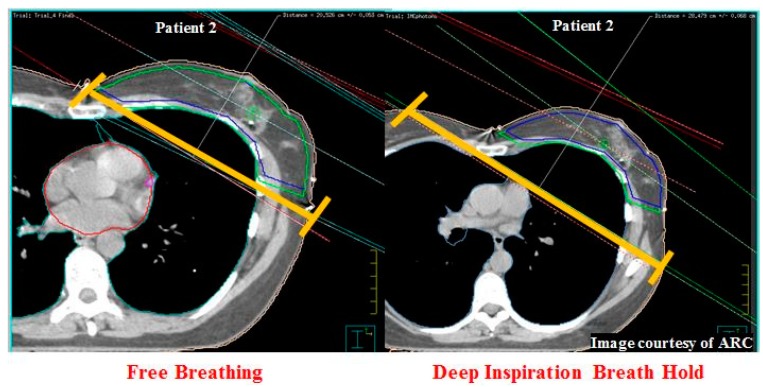
Demonstration of chest wall separation (CWS) (yellow) increasing from 28.4 cm in the FB plan (left) to 20.4 cm the DIBH plan (right) for patient 2 (multileaf collimator placement omitted). The heart (red), clinical target volume (blue) and planning target volume (green) are also demonstrated (Image Courtesy of ARC).

Central lung distance (CLD) is defined as the perpendicular distance from the posterior edge of the field border to the anterior chest wall (lung interior). The length is measured at the central axis of the tangential field in the digitally reconstructed radiograph (DRR) (Figure 3). Chest wall separation (CWS) is defined as the measurement between the most posterior field edges of the beam from the medial and lateral tangents of the non-diverging beam pair, measured at the centre of the field on the cranio-caudal axis (Figure 4) [29]. 

## 11. Statistical Analysis

Paired *t*-tests were used to statistically compare the dose distribution between the plans. This type of normal distribution analysis increases the statistical power of the study by reducing the variability that would be created if the samples were from different patients [30]. Unpaired t-tests were performed checking if both populations (FB and DIBH) had the same standard deviation. The *p*-value was investigated from a two-tailed test with a confidence interval of 95%. Correlations tests were performed using R^2^ to determine the selection criteria for left-sided breast cancer patients. Dose reduction of critical structures and anatomical parameters were compared between FB and DIBH plans. 

Availability of data and materials: Data supporting the results reported in the article are stored at the University of South Australia and are available upon request.

## 12. Results

### 12.1. Planning Volumes

The target coverage between the CT_DIBH_ and CT_FB_ plans was found comparable. Table 3 shows similar mean values and ranges of the relative dose covering a minimum of 90% of the PTV and 95% of the CTV. Treatment plans for both CT_DIBH_ and CT_FB_ were clinically acceptable and yielded the same coverage of the PTV and CTV.

### 12.2. Inter- and Intra-Observer Testing of LAD

There were no significant contouring differences identified between the three observers, therefore the dosimetric analysis results can be considered reproducible and valid. The LAD contours were found compatible between the inter- and intra-observer tests. The *p*-value was >0.05 for all measured similarity indices confirming minimal differences in LAD contouring between observers. 

### 12.3. Dosimetric Evaluation of DIBH

Dosimetric data collected from the CT_DIBH_ and CT_FB_ plans for all 20 patients are detailed in Table 4. 

### 12.4. Heart Dose

The fraction of heart volume receiving 10–30 Gy was consistently reduced by DIBH when analyzing the dose differences between CT_FB_ and CT_DIBH_ plans. Overall, the mean dose to the heart reduced from 2.7 Gy (FB) to 1.4 Gy (DIBH), resulting in a relative dose reduction of 1.3 Gy (45.7%). On average, heart D_mean_ for patients receiving a total dose of 50 Gy reduced by 1.3 Gy between plans, and 1.1 Gy for patients receiving a total dose of 42.4 Gy. 

**Figure 5:**
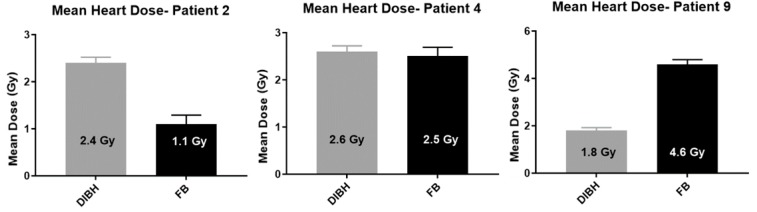
Variation of individual patient mean heart dose comparison results.

Figure 5 demonstrates two patients whose mean heart dose varies significantly between the techniques. Patient 9 receives a large dose reduction of 2.8 Gy using the DIBH technique but in comparison patient 2 and 4 receive no benefit.

However, there was no significant reduction of D_max_ between the techniques (average reduction of 5.1 Gy). On average, heart D_max_ for patients’ hearts receiving a total dose of 50 Gy reduced by 5.9 Gy between plans, and 3.1 Gy for patients receiving a total dose of 42.4 Gy. In three patients, the opposite effect was seen using the DIBH technique (patients 2, 6 and 11).

### 12.5. Left Anterior Descending Artery Dose

There was a significant reduction in the D_mean_ (7.5 Gy) received by the LAD for most patients using the DIBH technique. On average, LAD D_mean_ for patients receiving a total dose of 50 Gy reduced by 8.2 Gy between plans, and 5.7 Gy for patients receiving a total dose of 42.4 Gy. Similarly, the D_max_ received by the LAD was substantially reduced on average by 12.8 Gy. On average, LAD D_max_ for patients receiving a total dose of 50 Gy reduced by 16.7 Gy between plans, and 3.6 Gy for patients receiving a total dose of 42.4 Gy. Patients who demonstrated minimal changes in LAD D_mean_ also showed similar results for other recorded LAD parameters (patients 2, 5, 6 and 7). 

### 12.6. Total Lung Volume

The total lung volume (TLV) volume increased by 41.5% on average (1975 cm^3^) using the DIBH technique (2780 cm^3^ vs. 4755 cm^3^, *p* < 0.0001). Table 5 demonstrates this individual patient TLV difference from the CT_DIBH_ plan compared to the CT_FB_ plan as well as the respective CLD and CWS.

The relative reduction of CT_DIBH_ plan dose-volumes compared to CT_FB_ for TLV receiving V5, V10 and V20 was reduced by 12.4%, 13.6% and 13.8% respectively. This reduction across the TLV dose metrics was not statistically significant: *p* = 0.1508, *p* = 0.1501 and *p* = 0.1107. Therefore, subsequent findings focus on clinically significant reductions in OARs. 

### 12.7. Maximum Heart Distance

For all patients, MHD decreased (except for patient 2) using the DIBH technique and the heart was completely excluded from the beam in three of the 20 patients. 

### 12.8. Central Lung Distance

The shortest CLD observed across patients was identical, 1.6 cm, and belonged to the same patient (patient 3). The CLD did not extend beyond 3.9 cm in CT_FB_ plans (patient 10) and 5.6 cm for CT_DIBH_ plans (patient 2). Patient 2 had the largest increase in CLD across the plans (2.3 cm shown in Table 5), however across all measured ipsilateral lung (IL) and TLV dosimetric parameters demonstrated a negative effect for the DIBH technique, potentially suggesting that there is no relationship between the magnitude of dose reduction to OARs and CLD.

### 12.9. Chest Wall Separation

In contrast, this study found that the DIBH technique had limited effect on CWS distance as the average mean length was the same 23.1 cm across both plans (within statistical numbers) for the 20 patients. 

### 12.10. Correlation and Linear Regression of Analysed Dosimetric Data

The results of linear regression analysis that showed high correlation (i.e., high R^2^ value) are displayed in Figure S1. A correlation exists between mean heart dose difference and TLV measured in the FB plan and DIBH plan of Figures S1 and S2. Variables found to have substantial correlation are summarised in Table S1. The magnitude of dose reduction to cardiac structures was then correlated to the TLV taken in the CT_FB_ plan, creating a potential selection criteria for patients. 

### 12.11. Selection Criteria

Total lung volume measured in free breathing was identified as the strongest predictor across the most dose volume parameters observed in Table S1. The CWS measurement also showed correlation, but with a smaller number of patients (as compared to TLV). Therefore, only TLV was investigated as a predictive parameter to identify patients benefiting from DIBH. 

The TLV increased by 1975 cm^3^ on average using the DIBH technique, ranging widely from 1480 to 2625 cm^3^ (Table 5). Patients can be ranked in ascending order according to TLV difference between techniques (CT_DIBH_-CT_FB_) and allocated into three cohorts for the purposes of dose reduction analysis. Those who received a TLV reduction were described as (1) minimum ~1620 cm^3^ (range: 1480–1739 cm^3^), (2) medium ~1915 cm^3^ (range: 1820–1915 cm^3^) and 3) maximum ~2379 cm^3^ (range: 2165–2625 cm^3^) (Table 6).

## 13. Discussion

This study found that there was no significant difference in dose reduction to the D_max_ of the heart or V5-V20 of both the IL and TLV using DIBH. Both IL and TLV were significantly larger in CT_DIBH_ than CT_FB_ plans (*p* < 0.0001). CLD increased and MHD decreased using the DIBH technique, as expected; however, CWS showed minimal overall difference between the plans. Further analysis quantified a correlation between the TLV in CT_FB_ plan and the dose reduction to cardiopulmonary structures. This work is consistent with studies reviewed reporting a mean reduction in relative dose to OARs using the DIBH technique [3]. However, it is noted that the possible dose reduction effect in Figures S1, S2 and Table S1 could be impacted by the prescribed dose difference of the six patients receiving 42.4 Gy in 16 fractions rather than 50 Gy in 25 fractions (patients 4, 5, 6, 13, 16 and 18). While the majority of patients received the conventional prescription of 50 Gy in 25 fractions, the possible impact of the lower total treatment dose in this small sample size cannot be ruled out.

The current study confirms that not all patients receive benefit from the DIBH technique and patient outliers exist (e.g., patient 2). Most patients investigated benefited from the DIBH technique, or only a small difference between FB and DIBH was observed across cardiac dosimetric parameters (patients 1, 5, 6 and 7). These results varied equally across both techniques of breath hold (see Table 4) and therefore was not expected to impact on the dosimetric difference between plans.

Variation in chest shape can impact the cardiopulmonary dose that patients receive. In a minority of patients, the heart was seen to move with the anterior wall of the thorax. This chest shape impacts on the target volume and therefore can negate the cardiac sparing effects of the DIBH technique. The HI results did not indicate correlation with dosimetric reduction (except for D_mean_ heart dose). The lack of thoracic shape variation across the sample size could have determined this result. Variation in anatomical shape of patients (5 and 6) is believed to have resulted in a higher heart ΔD_mean_ for the ‘minimum benefit group’ than for the ‘maximum benefit group’. Similarly, outlying patient 2 reduces the entire results reported in Table 6 (the patient is in the ‘medium benefit group’ but receives none). 

Patients 2, 5, 6 and 7 received minimal to no relative dose reduction to the LAD in CT_DIBH_ plans across all measured parameters, indicating that the technique is not beneficial for all patients. This could be an anatomical parameter that informs selection for the technique as the chest shape dictates the anatomical location of the heart (the closer the anterior surface of the heart to the tangential beam the higher the LAD dose). The current study agrees with results reported by Hjelstuen et al. [15] and Vikstrom et al. [17] of a mean MHD reduction from 1.9 cm to 0.7 cm and 1.3 cm to 0.3 cm, using DIBH respectively. Shim et al. [31] also reported an increased CLD using DIBH, as expected, yielding similar results to this study. Their study linked the reduction in MHD and increased CLD distances to a reduction in irradiated lung volume. Studies by Vikstrom et al. [17] and Das et al. [29] state that an increase in CLD indicates a reduction in mean lung volume. Vikstrom et al. [17] correlated an increased CLD of 2.1 cm (FB) to 2.2 cm (DIBH) with the reduction in mean lung volume. As described, there was minimal statistically significant difference in CWS measured between plans. The only reviewed study that investigates CWS in DIBH patients is by Das et al. [29]. Contrary to this work, their study found patient’s CWS and D_max_ of the heart correlated with the size of the breast (R^2^ = 0.4178, *p* = 0.001), reiterating that anatomical parameters possibly provide a direct link to OAR dose reduction.

In contrast, Wang et al. [16] found a direct correlation between lung volume and the relative volume of the heart receiving 50 Gy (V50). Their study trialed screening patients through a rapid automated method of intensity modulated radiotherapy (IMRT) planning (using an automated script on the CT_FB_ plan) to deduce whether V50 < 10 cm^3^. The patient was considered to have unfavorable anatomy if their V50 (prescribed dose) exceeded 10 cm^3^ in the CT_FB_ plan and therefore underwent an additional DIBH scan. This method of patient selection was limited by the reliance on the rapid production of the CT_FB_ plan, resulting in 20 of the 53 participants being selected for DIBH treatment (indicating 33 achieved similar doses to cardiac structures using FB). 

As clearly demonstrated in this study, despite most patients receiving at least some dose reduction, not all patients benefit from the technique in all investigated parameters, and one patient in no parameters. While correlations are not strong, there is some established relationship between TLV and cardiac dosimetric parameters such as LAD. Due to the small sample size and weak correlation, we can conclude that not every patient benefits at a minimum. On average, Table 6 demonstrates the difference in TLV as a possible predictor of dose reduction across all measured LAD parameters and V20–V30 of the heart. Conversely, D_mean_ heart dose was observed as independent of TLV, possibly due to patient outliers (patients 1, 2 and 6). Based on the current study findings and alignment with previous literature discussed, this work suggests that a low dose scan could be taken in FB to assess TLV. The TLV is easily contoured as the density (i.e., Hounsfield units) is significantly different to surrounding tissue. At the time of this study, in South Australia, departments either select all left-sided breast cancer patients for treatment using DIBH or perform two high resolution CT scans and subsequent treatment plans (one in FB and the other in DIBH) to assess patient suitability. Therefore, it is proposed that patient dose can be potentially reduced if only one high resolution scan is performed in DIBH and a subsequent low dose scan in FB is taken to assess TLV, requiring less planning time and resources (see Figure S3 for a basic schematic representation of the possible department workflow).

The selection criteria suggested in this study would be applied prior to CT simulation and/or planning for DIBH, reducing CT dose to the patient and use of resources. Most patient selection criteria, employed in past studies, were based on post-planning results from a high-quality simulation scan in FB. Once the CT_FB_ plan was created and evaluated, the patient, identified using the selection criteria, was required to undertake a second high-quality scan in DIBH. This method delays the treatment start date, doubles high-dose CT exposure, and requires two plans (CT_FB_ and CT_DIBH_) to be generated, which results in more demand on resources. This is in agreement with a study by Tanguturi et al. [32] that identified a relationship between greater inspiratory lung volumes and larger dose reductions to cardiopulmonary structures when treating with the DIBH technique. Tanguturi et al. [32] suggested that all patients should be scanned in both DIBH and FB, as not every patient will experience a benefit from DIBH. Their multivariate analysis concluded that younger age, higher BMI and larger difference in inspiratory volume was associated with a greater reduction in D_mean_ of the heart using DIBH. Nissen, Appelt [25] investigated an extensive sample size (144) in their study, which reported little correlation between age and lung volume with heart dose (*p* = 0.002). They did deduce that lung volume reduced by 69 mL periodically for each 10 years of age, but this did not correlate with a dose reduction. 

There are two main differences between this study compared to previous research, which improve rigor of the results and allow for more generalization of the findings. First, the study included patients of both post-mastectomy and BCS, focusing on the clinical acceptability of the compared CT_DIBH_ and CT_FB_ plans. Secondly, inter- and intra-observer testing was performed to improve the reliability and generalization of the results. Other advantages of the study include addressing gaps in pre-existing literature such as improving methodology description. The same patient cohort was also used for both CT_FB_ and CT_DIBH_ plan generation unlike Nissen, Appelt [25]; as a result, paired t-tests could be performed. Importantly, no compromise was made to the patient’s treatment, as all data was retrospectively collected. Finally, this study aims to predict individual dosimetric reduction from the DIBH technique by creating a screening strategy for patients who will benefit the most, reducing radiation exposure and resource loss. 

Several measures were taken to ensure that the results of this study were reliable and valid; however, extenuating conditions existed due to the retrospective nature of the method. These include a small sample size, data collection methods and resource restrictions. Similar studies in the literature also had small sample sizes while justifying the significance of outcome. When the participant number is small and information on contouring methods is lacking, these studies are hard to reproduce, requiring further in-depth evaluation of ROIs. Ideally, larger sample sizes are needed for future retrospective studies. 

## 14. Conclusions

As clearly demonstrated in this study, not all patients benefit from the technique. A low dose CT scan could be taken in free breathing to measure the total lung volume. The patient can then be selected for either treatment, undertaking only one additional high-resolution scan for planning (abiding by the ALARA principle). This method requires less resources than the current method used and accounts for individual patient anatomy. This, in turn, results in patient-specific treatment minimizing the distress of patients.

Further prospective study is required where patients undertake two low dose CT scans, one in FB and the other in DIBH in order to assess TLV. The proposed selection criteria would be applied to confirm the results of this retrospective study.

## Figures and Tables

**Table 1:** Dose objectives used for plan evaluation.

Region of Interest	Target Goals	Variation Accepted
CTV	D95 ≥ 95%	D95 ≥ 90%
PTV	D90 ≥ 98%	D90 ≥ 95%
Heart	V10 < 10%	V10 < 15%
	V25 < 3%	V25 < 4%
	D_mean_ < 3 Gy	D_mean_ < 4 Gy
Ipsilateral lung	V30 < 12%	V30 < 15%
	V20 < 15%	V20 < 20%
	V10 < 255	V10 < 30%
	V5 < 30%	V5 < 50%
Total lung	V20 < 15%	V20 < 20%
	V10 < 20%	V10 < 25%
	V5 < 20%	V5 < 30%
Contralateral Breast	V3 < 3%	

Abbreviations: CTV: clinical target volume; PTV: planning target volume.

**Table 2:** Summary of patient data collected.

Parameters	Dosimetric Parameters	
Dosimetric	CTV	D95
	PTV	D90
	Ipsilateral lung	V5, V10, V20, V30 and volume
	Total lung	V5, V10, V20 and volume
	Heart	D_mean_, D_max_, V10, V20, V25 and V30
	LAD	D_mean_, D_max_, V20 and D0.2cm³
	Contralateral Breast	V3
Anatomical	Chest Wall Separation (CWS) Maximum Heart Distance (MHD) Central Lung Distance (CLD)	
Treatment	Type of deep inspiration breath hold Chemotherapy details Prescription Histological diagnosis Age Height Weight Comorbidities	

Abbreviations: CTV: clinical target volume; PTV: planning target volume.

**Table 3:** Difference in PTV D90 (%) and CTV D95 (%) across the FB and DIBH plans.

Target Coverage	CT_DIBH_		CT_FB_		*p*-value
	Mean Value (Range)	SD	Mean Value (Range)	SD	
**PTV D90 (%)**	88.5 (6.1–98.3)	23.0	89.8 (9.5–98.3)	19.3	>0.05 *
**CTV D95 (%)**	89.4 (4.5–98.7)	21.0	89.6 (5.7–98.9)	20.1	>0.05 *

Key: * Significant difference between plans if *p* < 0.05. Abbreviations: PTV: planning target volume; CTV: clinical target volume; SD: standard deviation; FB: free breathing; DIBH: deep inspiration breath hold.

**Table 4:** Dosimetric differences (Δ) from CT_FB_ - CT_DIBH_ plans for OAR for all 20 patients.

Patient No.	DIBH Device	Heart				LAD				Total Lung	
		ΔD_mean_ (Gy)	ΔD_max_ (Gy)	ΔV10 (%)	ΔV30 (%)	ΔD_mean_ (Gy)	ΔD_max_ (Gy)	ΔV20 (%)	ΔD0.2cm^3^ (Gy)	ΔV10 (%)	ΔV20 (%)
1	Voluntary BH §	1.9	8.4	5.8	3.0	15.1	33.7	38.0	42.5	1.0	1.4
2	Voluntary BH §	−1.3	−25.4	−2.6	0.0	−3.9	−19.6	−8.0	−12.4	−5.9	−5.8
3	ABC §	1.1	11.5	3	2.0	8.7	11.7	22.0	24.1	0.3	0.6
4	Voluntary BH †	−0.1	2.7	2.8	1.0	11.7	4.9	44.0	19.5	13.4	2.7
5	ABC †	1.5	−1.1	3.9	3.0	−3.5	−22.1	−10.0	−2.3	0.1	0.2
6	Voluntary BH †	1.2	−4.1	0.1	0.0	0.6	−3.3	−5.0	−4.0	0.9	0.9
7	ABC §	0.6	14.7	1.5	1.0	0	−5.5	0	−1.0	2.8	2.9
8	Voluntary BH §	1.5	2.5	5.1	1.0	12.0	4.6	52.0	16.9	−1.9	−1.6
9	Voluntary BH §	2.8	7.8	5.3	3.0	9.6	34.6	25.0	37.9	−1.1	−0.9
10	ABC §	1.9	10.5	3.5	2.0	12.2	38.1	31.0	39.6	4.1	4
11	ABC §	1.6	2.3	−15.7	3.0	11.0	3.2	25.0	11.7	3.9	4.1
12	ABC §	1.7	33.0	4.5	3.0	11.0	42.6	29.0	39.6	3.6	3.3
13	Voluntary BH †	0.2	13.8	1.4	0.0	2.8	19.9	2.0	9.4	3.9	3.5
14	Voluntary BH §	1.3	4.8	3.1	2.0	1.0	6.6	0	1.6	2.1	2.2
15	ABC §	1.0	17.4	2.9	1.0	4.5	27.4	10.0	18.0	−2.2	−1.7
16	Voluntary BH †	2.1	6.6	6.4	4.0	17.1	22.8	53.0	30.1	1.1	1.6
17	Voluntary BH §	1.2	−1.6	4.9	3.0	9.7	28.1	34.0	35.5	−3.2	−2
18	ABC †	1.7	0.7	6.1	4.0	5.4	−0.6	17.0	5.1	5.0	4.8
19	ABC §	1.5	0.0	3.9	2.0	12.5	19.1	34.0	34.3	−2.3	−1.5
20	ABC §	1.8	−2.6	5.9	3.0	12.3	9.2	41.0	22.1	1.3	1.1
**Mean**		1.3 (1.3 §, 1.1 †)	5.1 (5.9 §, 3.1 †)	2.6 (2.2 §, 3.5 †)	2.0 (2.1 §, 2.0 †)	7.5 (8.2 §, 5.7 †)	12.8 (16.7 §, 3.6 †)	21.7 (23.8 §, 16.8 †)	18.4 (22.2 §, 9.6 †)	1.3 (0.2 §, 4.1 †)	1 (0.4 §, 2.3 †)

**Key:** §: patient prescribed 50 Gy dose in 25 fractions; †: patient prescribed 42.4 Gy dose in 16 fractions. Grey indicates no dosimetric benefit from DIBH. Abbreviations: BH: breath hold; ABC: Active Breathing Coordinator.

**Table 5:** CLD, TLV, MHD and CWS from CT_FB_ and CT_DIBH_ plans for individual patients.

Patient No.	CLD (cm)			TLV (cm^3^)			MHD (cm)			CWS (cm)		
	CT_FB_	CT_DIBH_	Difference Δ	CT_FB_	CT_DIBH_	Difference Δ	CT_FB_	CT_DIBH_	Difference Δ	CT_FB_	CT_DIBH_	Difference Δ
1	2.4	2.3	−0.1	3207	5076	1869	1.8	0.6	1.2	22.5	20.5	2
2	3.3	5.6	2.3	4788	6608	1820	0.0	0.0	0.0	20.4	28.4	−8
3	1.6	1.6	0	1712	3443	1731	1.2	0.6	0.6	21.3	21.0	0.3
4	2.4	2.5	0.1	2769	4714	1945	1.0	0.5	0.5	20.4	19.4	1
5	2.5	2.7	0.2	3330	4922	1592	1.7	1.4	0.3	22.0	22.1	−0.1
6	2.2	2.6	0.4	2840	4579	1739	2.3	1.0	1.3	22.1	19.1	3
7	2.9	3.0	0.1	3186	5765	2579	1.1	0.0	1.1	21.6	22.4	−0.8
8	2.1	2.4	0.3	2275	3755	1480	1.5	1.3	0.2	29.1	28.8	0.3
9	2.1	3.1	1	2336	4277	1941	1.9	1.2	0.7	21.7	21.8	−0.1
10	3.9	4.1	0.2	2783	4354	1571	3.1	2.2	0.9	23.5	21.3	2.2
11	2.0	3.3	1.3	2520	4461	1941	1.7	1.4	0.3	26.2	27.6	−1.4
12	2.2	1.8	−0.4	2335	4714	2379	1.6	0.1	1.5	22.8	22.2	0.6
13	2.9	2.4	−0.5	3341	5320	1979	1.6	0.8	0.8	22.6	21.4	1.2
14	2.4	2.2	−0.2	2561	4166	1605	1.3	0.8	0.5	22.3	21.8	0.5
15	2.1	2.2	0.1	2271	3893	1622	2.1	0.8	1.3	22.6	22.6	0
16	2.6	2.9	0.3	2730	5214	2484	2	0.7	1.3	25.7	25.8	−0.1
17	1.6	2.2	0.6	2698	4863	2165	1.2	1.0	0.2	21.9	22.8	−0.9
18	3.4	3.0	−0.4	2600	4787	2187	1.9	0.6	1.3	21.1	19.7	1.4
19	1.6	1.8	0.2	2767	5005	2238	1.6	0.5	1.1	26.9	26.6	0.3
20	2.5	2.5	0	2556	5181	2625	3.8	3.7	0.1	26.3	27.0	−0.7
Mean	2.4	2.7	0.3	2780	4755	1975	1.7	1.0	0.8	23.1	23.1	0

Key: Grey indicates no dosimetric benefit from DIBH. Abbreviations: CLD: central lung distance; TLV: total lung volume; MHD: maximum heart distance; CWS: chest wall separation.

**Table 6:** Individual total lung volume between FB and DIBH plans ranked and grouped in ascending order.

Benefit Group	Patient	Δ TLV (cm^3^)	Heart Relative Reduction					LAD Relative Reduction		
			Δ D_mean_ (Gy)	Δ V10 (%)	Δ V20 (%)	Δ V25 (%)	Δ V30 (%)	Δ D_mean_ (Gy)	Δ D0.2cm^3^ (Gy)	Δ V20 (%)
**Minimum**	8	1480	1.5	5.1	4	2.1	1	12.0	16.9	52
	10	1571	1.9	3.5	3	2.5	2	12.2	39.6	31
	5	1592	1.5	3.9	3	3.1	3	−3.5	−2.3	−10
	14	1605	1.3	3.1	3	2.1	2	1.0	1.6	0
	15	1622	1	2.9	2	1.8	1	4.5	18.0	10
	3	1731	1.1	3	3	1.8	2	8.7	24.1	22
	6	1739	1.2	0.1	−1	−0.3	0	0.6	−4.0	−5
	Mean	1620	1.3	3.0	2.4	1.8	1.5	5.1	13.4	14.2
**Medium**	2	1820	−1.3	−2.6	−1	−0.4	0	−3.9	−12.4	−8
	1	1869	1.2	5.8	5	3.9	3	15.1	42.5	38
	9	1941	1.2	5.3	4	3.8	3	9.6	37.9	25
	11	1941	1.2	−15.7	3	2.7	3	11	11.7	25
	4	1945	1.2	2.8	2	1.5	1	11.7	19.5	44
	13	1979	1.2	1.4	1	0.3	0	2.87	9.41	2
	Mean	1915	0.7	−0.5	2.3	1.9	1.6	7.7	18.1	21
**Maximum**	17	2165	1.2	4.9	3	3.3	3	9.7	35.5	34
	18	2187	1.2	6.1	4	4.2	4	5.4	5.11	17
	19	2238	1.2	3.9	3	2.7	2	12.5	34.3	34
	12	2379	1.2	4.5	3	2.9	3	11.0	39.6	29
	16	2484	1.2	6.4	5	4.5	4	17.1	30.1	53
	7	2579	1.2	1.5	1	0.8	1	0	−1	0
	20	2625	1.2	5.9	5	3.4	3	12.3	22.1	41
	Mean	2379	1.2	4.7	3.4	3.1	2.8	9.7	23.7	29.7

Abbreviations: Δ: relative reduction; TLV; total lung volume.

## Supplementary Materials

The following are available online at https://www.mdpi.com/2072-6694/11/2/259/s1, Figure S1: Reduction in dosimetric parameters correlated with patient anatomical parameters. (A) Correlation between the mean heart dose difference (ΔD_mean_) and total lung volume (TLV) for CT_DIBH_ (left) and CT_FB_ (right) plans. (B) Correlation between the difference in volume of the heart receiving 20 Gy (ΔV20) and total lung volume (TLV) for CT_FB_ (left) and CT_DIBH_ (right) plans. (C) Correlation between the mean LAD dose difference (ΔD_mean_) and total lung volume (TLV) for CT_FB_ (left) and CT_DIBH_ (right) plans. (D) Correlation between the difference in dose received by 0.2 cm^3^ of the LAD (Δ D0.2cm^3^) and total lung volume (TLV) for CT_FB_ (left) and CT_DIBH_ (right) plans. (E) Correlation between the difference in the LAD volume receiving 20 Gy (ΔV20) and total lung volume (TLV) for CT_FB_ (left) and CT_DIBH_ (right) plans, Figure S2: (A) Linear regression of the mean heart dose difference (ΔD_mean_) and chest wall separation difference (ΔCWS) between CT_FB_ and CT_DIBH_ plans. (B) Linear regression of the mean heart dose difference (ΔD_mean_) and Haller Index, Table S1: Summary of reduction in dosimetric parameters correlated with patient anatomical parameters, Figure S3: Potential treatment scheme compared to the treatment scheme in South Australia at the time of this study.
