# Supplementary material for: A Retrospective Dosimetric Study of Radiotherapy Patients with Left-Sided Breast Cancer; Patient Selection Criteria for Deep Inspiration Breath Hold Technique
Source: Cancers (Basel). 2019 Feb 22;11(2):259. doi: 10.3390/cancers11020259 (PMC6406815; doi:10.3390/cancers11020259)
Supplement: Supplementary file 1 [file cancers-11-00259-s001.pdf]

Supplementary Materials: A Retrospective Dosimetric Study of Radiotherapy Patients with Left-Sided Breast Cancer; Patient Selection Criteria for Deep Inspiration Breath Hold Technique

Mikaela Dell'Oro, Eileen Giles, Amy Sharkey, Martin Borg, Caroline Connell and **Eva Bezak**

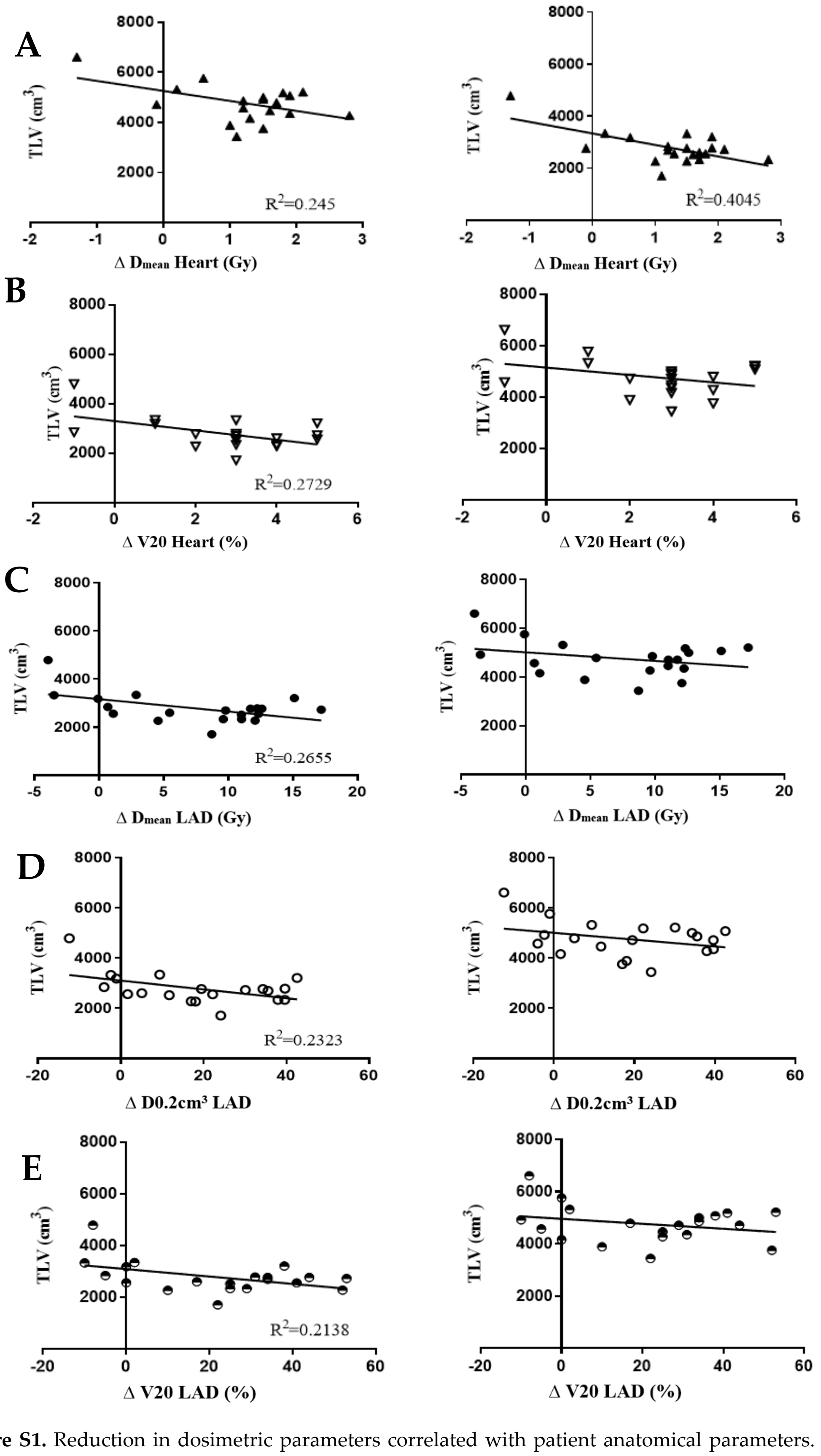

Figure S1. Reduction in dosimetric parameters correlated with patient anatomical parameters. (**A**) Correlation between the mean heart dose difference (ΔD_{mean}) and total lung volume (TLV) for CT_{DIBH} (left) and CT_{FB} (right) plans. (**B**) Correlation between the difference in volume of the heart receiving 20 Gy ($\Delta V20$) and total lung volume (TLV) for CT_{FB} (left) and CT_{DIBH} (right) plans. (**C**) Correlation between the mean LAD dose difference (ΔD_{mean}) and total lung volume (TLV) for CT_{FB} (left) and

CTDIBH (right) plans. (**D**) Correlation between the difference in dose received by 0.2 cm³ of the LAD (Δ D0.2cm³) and total lung volume (TLV) for CTFB (left) and CTDIBH (right) plans. (**E**) Correlation between the difference in the LAD volume receiving 20 Gy (ΔV20) and total lung volume (TLV) for CTFB (left) and CTDIBH (right) plans.

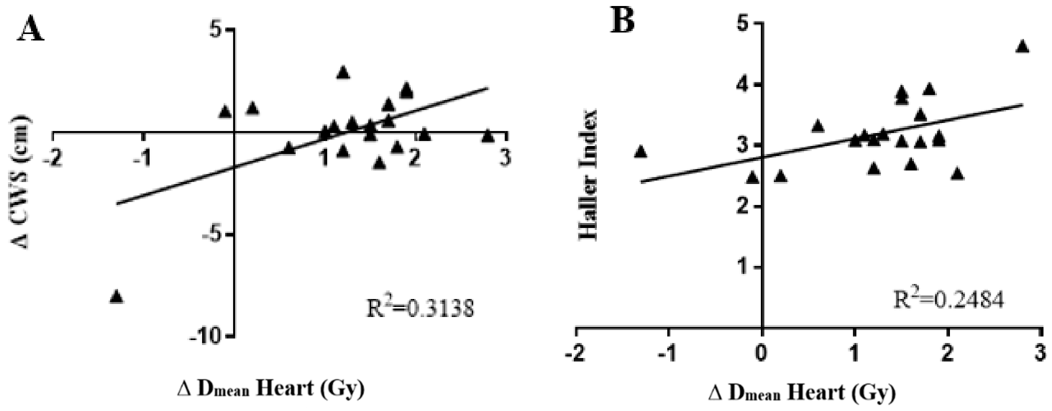

Figure S2. (**A**) Linear regression of the mean heart dose difference (ΔDmean) and chest wall separation difference (ΔCWS) between CTFB and CTDIBH plans. (**B**) Linear regression of the mean heart dose difference (ΔDmean) and Haller Index.

Table S1. Summary of reduction in dosimetric parameters correlated with patient anatomical parameters.

		Δ CLD	CWS in CT_{FB} Plan	Δ CWS	MHD in CT_{FB} Plan	Haller Index	TLV in CT_{DIBH} Plan	TLV in CT_{FB} Plan
Heart	ΔD_{mean}			0.3138*	0.397*	0.2484*	0.245*	0.4045*
	ΔV10	0.2906*						
	ΔV20		0.2047*					0.2729*
	ΔV25							
	ΔV30							
LAD	ΔD_{mean}		0.2799*					0.2655*
	$\Delta D0.2cm^3$							0.2323*
	ΔV20		0.3146*					0.2138*

Key: *Significant difference irrespective of magnitude. Abbreviations: Δ: relative reduction; CLD: central lung distance; MHD: maximum heart distance; CWS: chest wall separation: TLV; total lung volume.

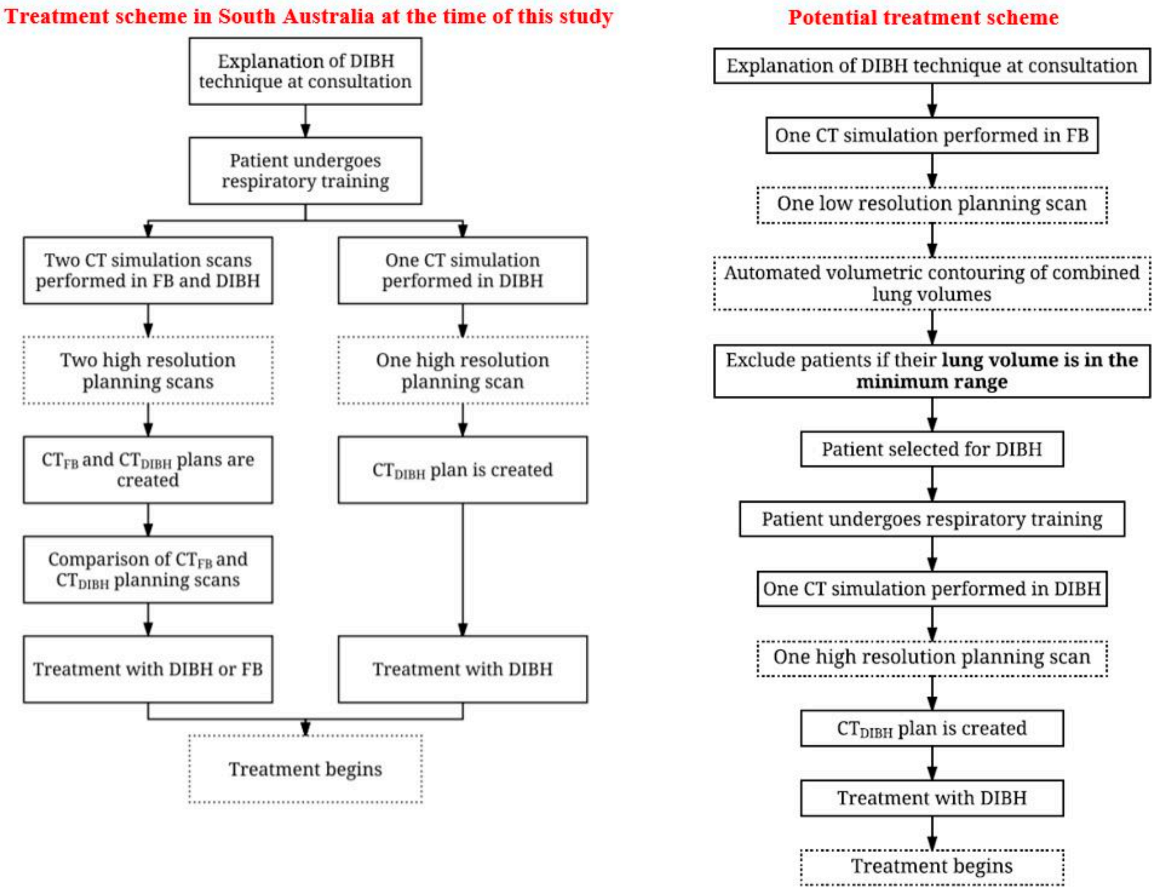

Figure S3. Potential treatment scheme compared the treatment scheme in South Australia at the time of this study.
